# Anoxia-adapted cyanobacteria in a marine blue hole

**DOI:** 10.1128/aem.02576-25

**Published:** 2026-02-23

**Authors:** Zhuobo Li, Hongxi Zhang, Taoshu Wei, Lisheng He, Yong Wang

**Affiliations:** 1 Institute for Ocean Engineering, Shenzhen International Graduate School, Tsinghua University 12442, Shenzhen, China; 2 Institute of Deep Sea Science and Engineering, Chinese Academy of Sciences 383875 , Sanya, China; 3 Shenzhen Key Laboratory of Advanced Technology for Marine Ecology, Shenzhen International Graduate School, Tsinghua University 118351 , Shenzhen, China; University of Delaware, Lewes, Delaware, USA

**Keywords:** evolution, anoxia-adapted, cyanobacteria

## Abstract

Vertical transmission of marine particles brings ocean surface cyanobacteria into the deep ocean, where heterotrophic cyanobacterial lineages probably evolve to adapt to new environments even in oxygen-depleted zones. At present, active cyanobacteria have rarely been reported in dark and anoxic water columns in the deep sea. In this study, we recovered three metagenome-assembled genomes of cyanobacteria from the Yongle blue hole located in the South China Sea, two of which were actively transcribed in a dark, anoxic environment at 250 m depth, through integrated metagenomic and metatranscriptomic analyses of water samples from 21 stratified depths collected using *in situ* microbial fixation and filtration. These anoxia-adapted cyanobacteria were phylogenetically approximate to the sponge cyanobacterial symbionts, while the genomic features showed similarities with both free-living and sponge symbiotic counterparts. They exhibit genomic features shared with symbiotic lineages, including loss of substrate utilization, biosynthesis pathways, DNA repair, and circadian regulation. Conversely, they retain selected metabolic characteristics of free-living lineages, including phenylalanine biosynthesis and phosphoserine metabolism. Additionally, the discovery of taurine transport proteins in the genomes suggests the potential for organic sulfur uptake from the environment. Altogether, these findings reveal a distinct genomic configuration in cyanobacteria inhabiting a permanently dark and anoxic marine system, characterized by the retention of oxygen-dependent metabolic potential alongside sustained transcriptional suppression under *in situ* conditions. This study provides new insights into the ecological persistence and evolutionary adaptation of cyanobacteria under long-term oxygen limitation.

**IMPORTANCE:**

We report metabolically active cyanobacteria thriving in darkness and oxygen deprivation at 250 m depth in the ocean. Genomics results show these microbes share evolutionary roots with sponge cyanobacterial symbionts but developed unique adaptations for anoxic and sulfidic environments. Strikingly, they retain photosynthesis genes as genomic remnants (with no detected transcription) while losing genes critical for environmental stress responses, including DNA repair, osmotic regulation, and circadian control, suggesting a potential evolutionary connection to symbiotic relatives. Crucially, they maintain metabolic autonomy via phenylalanine biosynthesis and light-independent serine biosynthesis, exhibiting traits absent in most symbionts. This demonstrates how cyanobacteria adapt to anoxic environments through targeted genome reduction, revealing novel survival strategies in oxygen-depleted oceans and providing a research case for microbial resilience during marine deoxygenation.

## INTRODUCTION

Cyanobacteria are an ancient lineage of photosynthetic prokaryotes that play a key ecological role, such as oxygen production, nitrogen fixation, and carbon flux (1, 2). Among them, *Synechococcus* are dominant cyanobacteria in the ocean surface. They are autotrophic and oxygenic phototrophs and are therefore the main source of primary production for the ecosystem of oligotrophic, pelagic marine waters. Free-living *Synechococcus* were distributed between the surface layer and the bottom of the euphotic zone (3). Multiple lineages of *Synechococcus* were discovered to spread in global coastal and open oceans, even in polar regions (4, 5). Aside from photosynthesis, *Synechococcus* are able to degrade peptides and other organic matter (3, 6). In dark anoxic conditions, cyanobacterium *Oscillatoria* can ferment glycogen and other carbon storage for energy temporarily (7–9). This metabolic flexibility also facilitates symbiotic lifestyles, allowing cyanobacteria to thrive in nutrient-rich environments with low light density. Previous studies have revealed that sponge-associated bacterial communities possess metabolic pathways involved in carbon fixation, synthesis of B vitamins, taurine metabolism, sulfate oxidation, and nitrogen metabolism (10). *Candidatus* Synechococcus spongiarum is known as a sponge symbiont with specific adaptation strategies to cope with low light density in a sponge host (11). This obligate symbiont exhibits genome reduction (11), similar to other bacterial sponge symbionts, like *Candidatus* Endohaliclona renieramycinifaciens (12). Previously, a strain of phycoerythrin-rich *Synechococcus* sp. was discovered in the Black Sea and has been indicated to reactivate in dark and anoxic conditions (13). Given the coincidence with the Chl *a* signal in the deep Black Sea, the *in situ* activity of the *Synechococcus* sp. remains a question. Together, there are scarce reports of heterotrophic anaerobic *Synechococcus* in permanently dark, anoxic environments. The origin and ecological contribution of the anaerobic *Synechococcus* in the dark ocean are yet to be determined.

The Yongle blue hole (YBH) located in the Xisha Islands of the South China Sea (SCS) exhibits oxygen gradients, with the deep waters over ~100 m depth being a permanent dark and anoxic zone (14). The light can penetrate into different depths in different seasons, resulting in variant levels of the oxic-anoxic interface of YBH (15). From ~100 m to the bottom at 300.89 m, YBH is filled with methane- and sulfide-rich water without light (16). Our recent metagenomics and metatranscriptomics study has predicted the microbial source of methane and how the methane was consumed in the upper water column of YBH (16). Recent viral analyses further indicate strong niche differentiation across the oxic-anoxic gradient of the YBH (17). A higher abundance of *Synechococcus* has been discovered in the hole, encompassing both the illuminated, oxygen-rich upper layer and the oxygen-deprived habitats (18). However, the rapid light attenuation may limit the growth of *Synechococcus* in the upper layers (19). The lifestyle of *Synechococcus* has been predicted through functional gene prediction, which is primarily photoautotrophic in light and oxygenated environments (18). However, the survival mechanisms employed by *Synechococcus* inhabiting dark, anoxic environments are yet to be explored. The Amberjack blue hole in the Gulf of Mexico hosts *Synechococcus* as well, which is only present in the shallow waters (20). Nevertheless, our current understanding of the survival strategies of *Synechococcus* in deep waters remains very limited. Conducting a genomic characterization of the *Synechococcus* inhabiting the anoxic deep waters of YBH may provide valuable insights into the origin and adaptive mechanisms of the novel species.

In this study, we report the draft genomes and transcriptomes of one *Synechococcus* from the oxic water and two anoxia-adapted *Synechococcus* from the anoxic water in YBH. We have compared these genomes with those from the extracellular, obligate symbiont species “*Ca*. Synechococcus spongiarum” and with free-living cyanobacterial counterparts to search for specific genomic traits for adaptation. Given that “*Ca*. Synechococcus spongiarum” represents a lineage known for genome reduction in light-limited sponge microhabitats, its phylogenomic proximity to the YBH anoxia-adapted *Synechococcus* raises the possibility that these populations might share a symbiotic or symbiont-derived evolutionary history. Our study provides the metatranscriptomic evidence for actively transcribing *Synechococcus*-like cyanobacteria in permanently dark, anoxic deep waters and identifies a distinct genomic pattern, characterized by the loss of stress-response genes together with the retention of key free-living metabolic pathways. This combination supports long-term adaptation to anoxic, sulfidic environments and suggests a possible evolutionary link to sponge-associated lineages.

## RESULTS

### Genomic features and transcriptomic level of cyanobacteria

**Fig 1:**
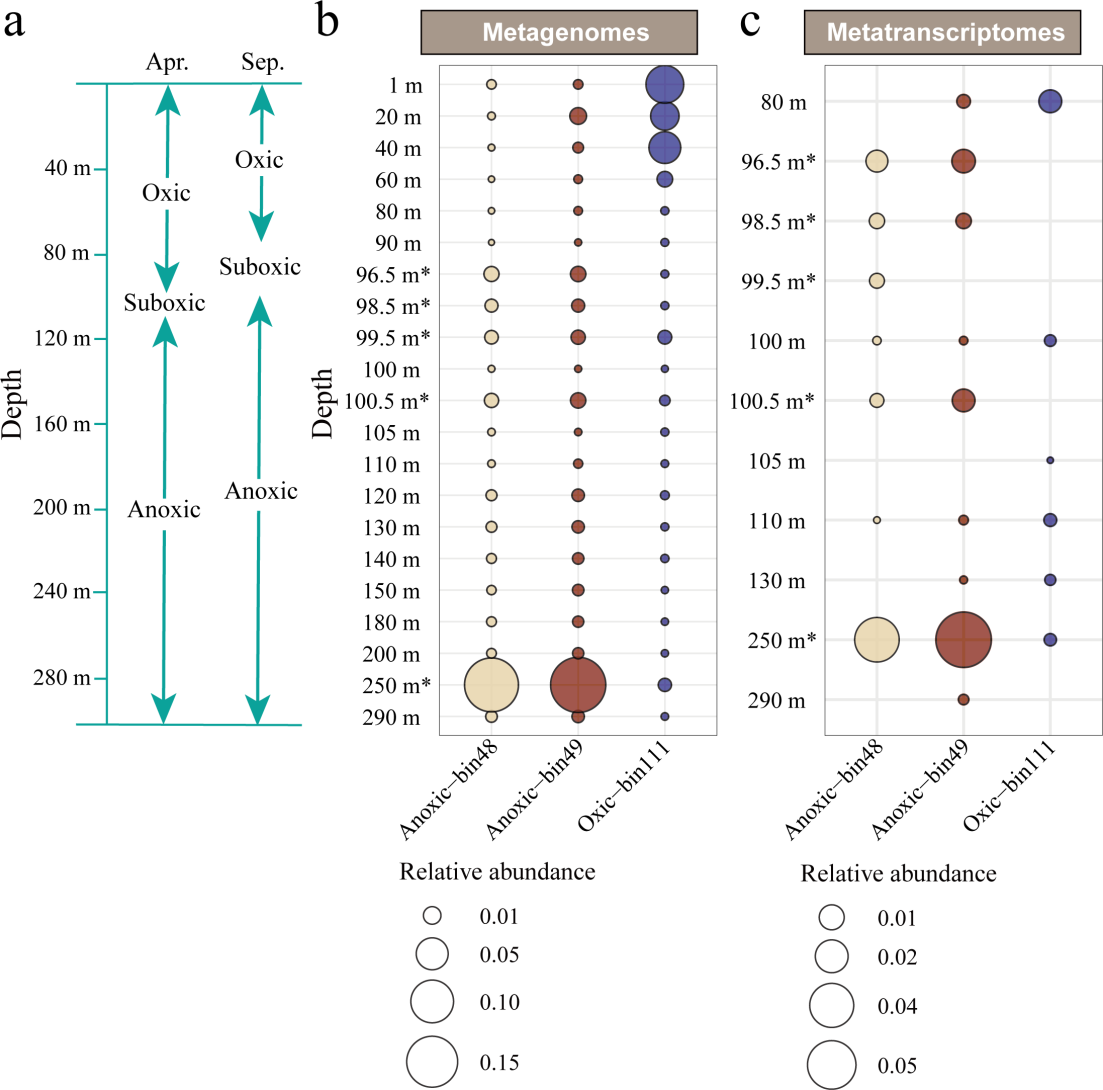
Relative abundance of *Synechococcus* MAGs in metagenomes and metatranscriptomes. (**a**) The division of oxic, suboxic, and anoxic zones in YBH. (**b**) The relative abundance of three *Synechococcus* MAGs across 21 metagenomes. (**c**) The relative abundance of three *Synechococcus* MAGs across 11 metatranscriptomes. Oxic-bin111 represents a *Synechococcus* genome reconstructed from the oxic layer, while Anoxic-bin48 and Anoxic-bin49 are two closely related Synechococcus genomes recovered from the anoxic layer. The samples marked with an asterisk (*) at the depth were collected in September, while the rest were collected in April 2021.

We obtained a total of 162.48 billion base pairs (Gbp) metagenomes and 23.61 Gbp metatranscriptomes for the 21 samples (Table S1). After assembly, genome binning obtained 319 metagenome-assembled genomes (MAGs) (>50% completeness and <10% contamination), associated with their relative abundances in proxy of percentage of metagenomic and metatranscriptomic reads mapped on the MAGs (Table S2 and S3). Three MAGs (bin48, bin49, and bin111) affiliated with the *Synechococcus* genus of cyanobacteria were binned from the metagenomes, with the genome size ranging from 1.30 to 1.92 Mbp. The completeness of bin48 and bin49 genomes was 94.43% and 96.88%, respectively (Table S4). However, bin111 was relatively lower in completeness (70.43%). The relative abundance and transcriptional level of the three *Synechococcus* species were estimated based on the percentage of the reads mapped on the three individual MAGs from the 21 metagenomes and 11 metatranscriptomes, respectively (Fig. 1). The result showed that the bin111 had the highest abundances in the metagenomes of 1–40 m depths (0.04%–0.07% read recruitment), ranking among the top 5% of all MAGs in these oxic layers (Fig. S1 and S2a), and exhibited transcriptional activity at 80 m depth (0.01%, top 5% of all MAGs, Fig. S2b) (hereafter Oxic-bin111). On the other hand, both bin48 and bin49 genomes showed the highest abundance (0.17% [33rd of 319] and 0.18% [30th of 319] read recruitment, respectively) at the 250 m depth within the anoxic layer (hereafter, Anoxic-bin48 and Anoxic-bin49). The two genomes also exhibited transcriptional activity at this depth with recruitment of 0.04% (82nd of 319) and 0.07% (57th of 319) of the transcriptomic reads, respectively. Independent validation using 16S miTag (16S rRNA gene fragments for V4 region) data further supported these patterns (Fig. S2c). Cyanobacterial sequences related to Oxic-bin111 were dominant in the 1–60 m layers, while those affiliated with the anoxia-adapted lineages were predominant at 250 m. Based on these findings, the *Synechococcus* in the surface oxic layer inhabits well-illuminated and oxygen-rich shallow waters, while the remaining two, represented by the recovered MAGs from the anoxic layers, are capable of thriving in the dark and light-deprived water.

### Phylogenomics analysis of cyanobacterial genomes

**Fig 2:**
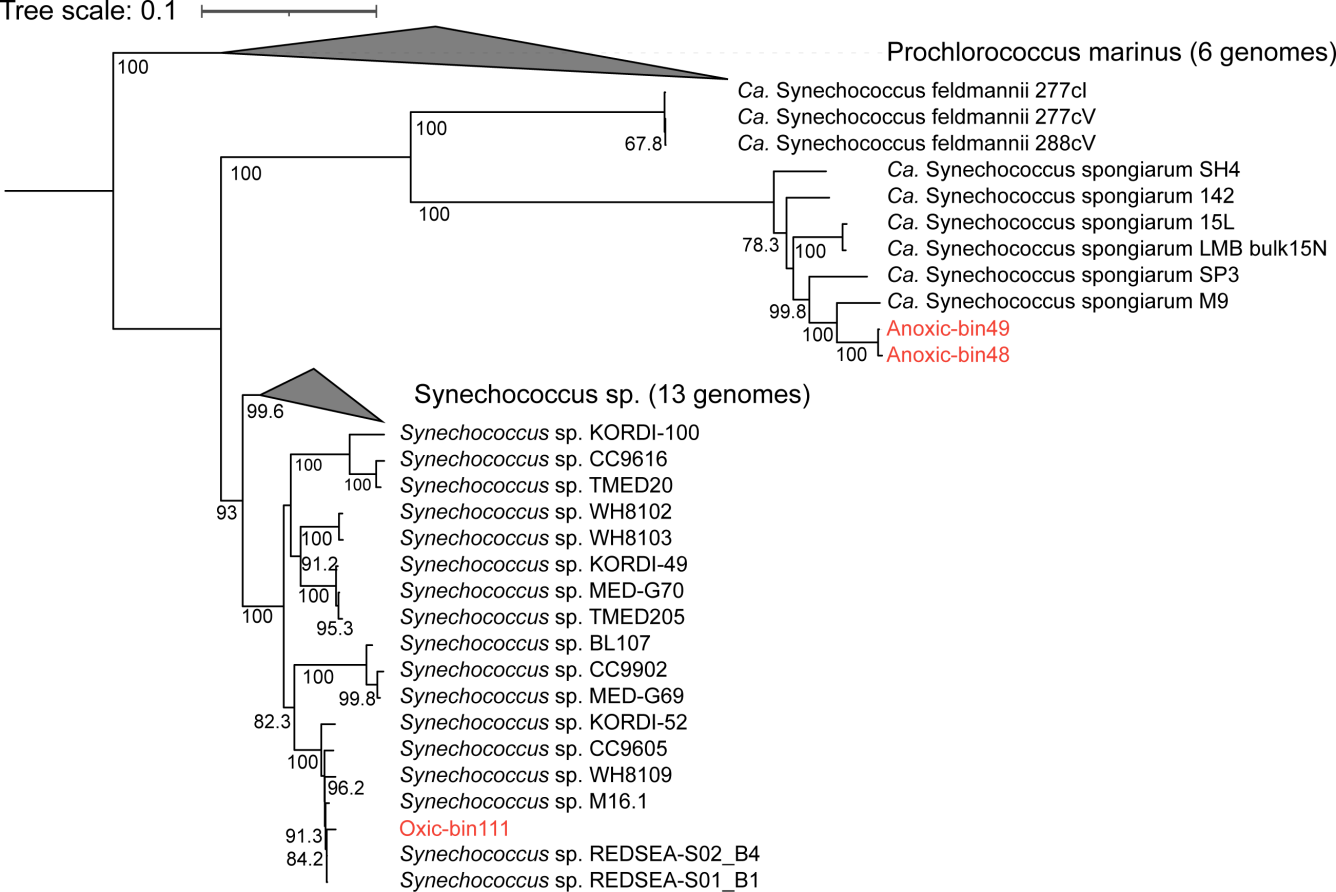
Maximum-likelihood phylogenomics tree of cyanobacteria MAGs. The maximum likelihood phylogenetic tree of the three cyanobacteria genomes from this study, with 43 conserved proteins from 50 reference genomes. The cyanobacteria genomes from this study are indicated in red.

To examine the taxonomic positions of these MAGs, we performed phylogenomics analysis using conserved proteins encoded by the three genomes (Fig. 2). The Oxic-bin111 showed a closer phylogenetic relationship with free-living *Synechococcus* sp., with the closest neighbors being *Synechococcus* sp. REDSEA-S01_B1 and *Synechococcus* sp. REDSEA-S02_B4 were collected from the Red Sea. On the other hand, Anoxic-bin48 and Anoxic-bin49 were more closely related to the sponge-associated cyanobacterium, “*Ca*. Synechococcus spongiarum.” The closest strain is *Ca*. Synechococcus spongiarum M9 hosted by the sponge *Theonella swinhoei* in the SCS (21). Additionally, we calculated average nucleotide identity (ANI) between the 3 MAGs and the 31 reference genomes (Fig. S3). Anoxic-bin48 and Anoxic-bin49 showed an ANI value of 86.8% and 86.9%, respectively, with *Ca*. Synechococcus spongiarum M9. In light of these results, the two MAGs from the anoxic layer probably share more genetic features with the sponge-associated cyanobacteria. The ANI between Oxic-bin111 and S. REDSEA-S01_B1 was 98.2%, suggesting a high affinity of Oxic-bin111 with known *Synechococcus* strains.

### Differences in functional genes of anoxia-adapted *Synechococcus*

**Fig 3:**
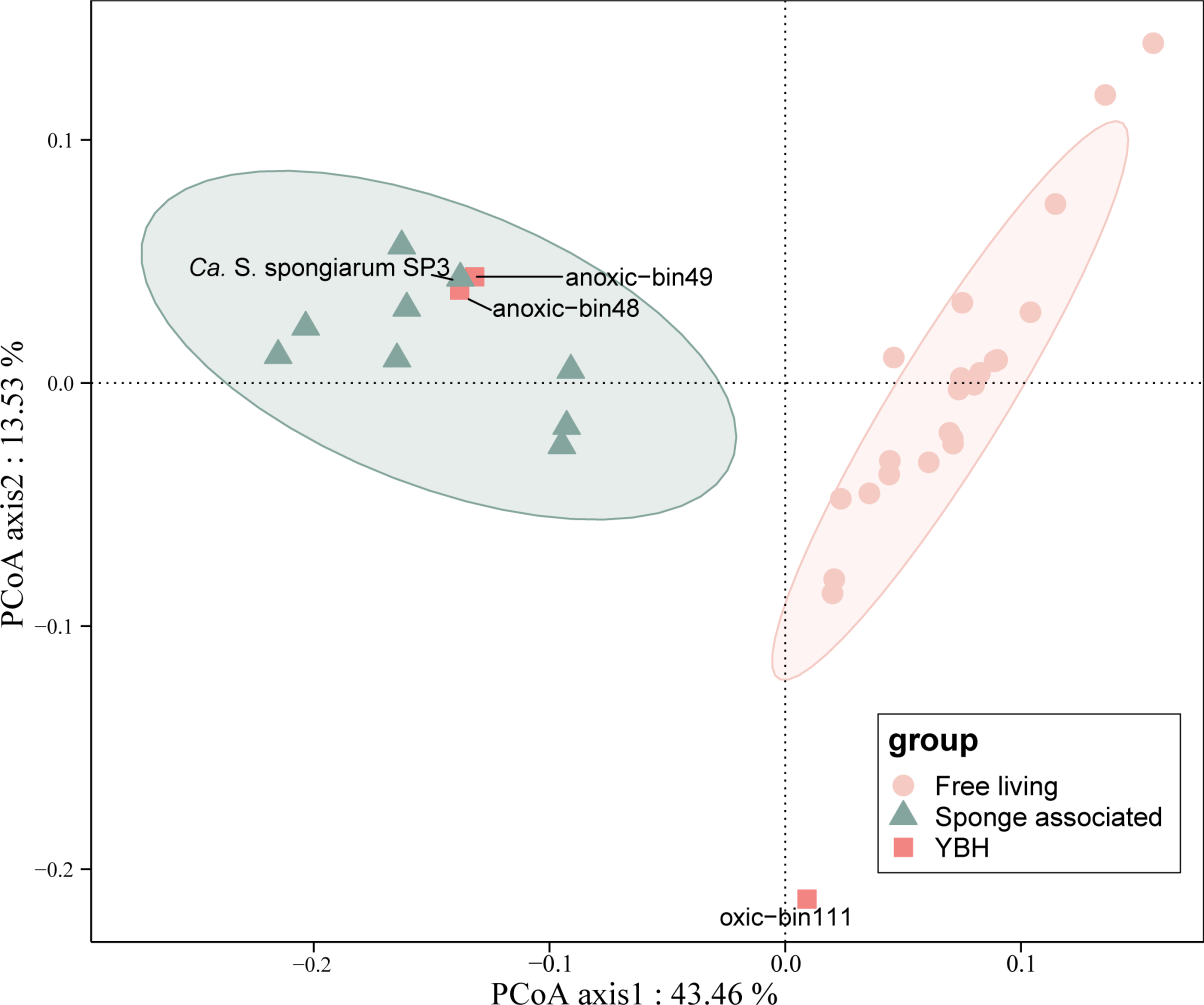
PCoA analysis of the relative abundance of functional genes based on COG annotation. The genomes of free-living and sponge-associated groups are listed in Table S4.

Based on the annotation results against the COG database (22), PCoA was conducted to demonstrate the functional divergence between the cyanobacterial genomes. The two anoxia-adapted cyanobacteria in YBH clustered together with the sponge-associated cyanobacteria (Table S5 and Fig. 3). In contrast, the Oxic-bin111, possibly due to their lower genome completeness or independent evolution in the semi-isolated YBH (Table S4), was distantly associated with the genomes of other free-living cyanobacteria.

**Fig 4:**
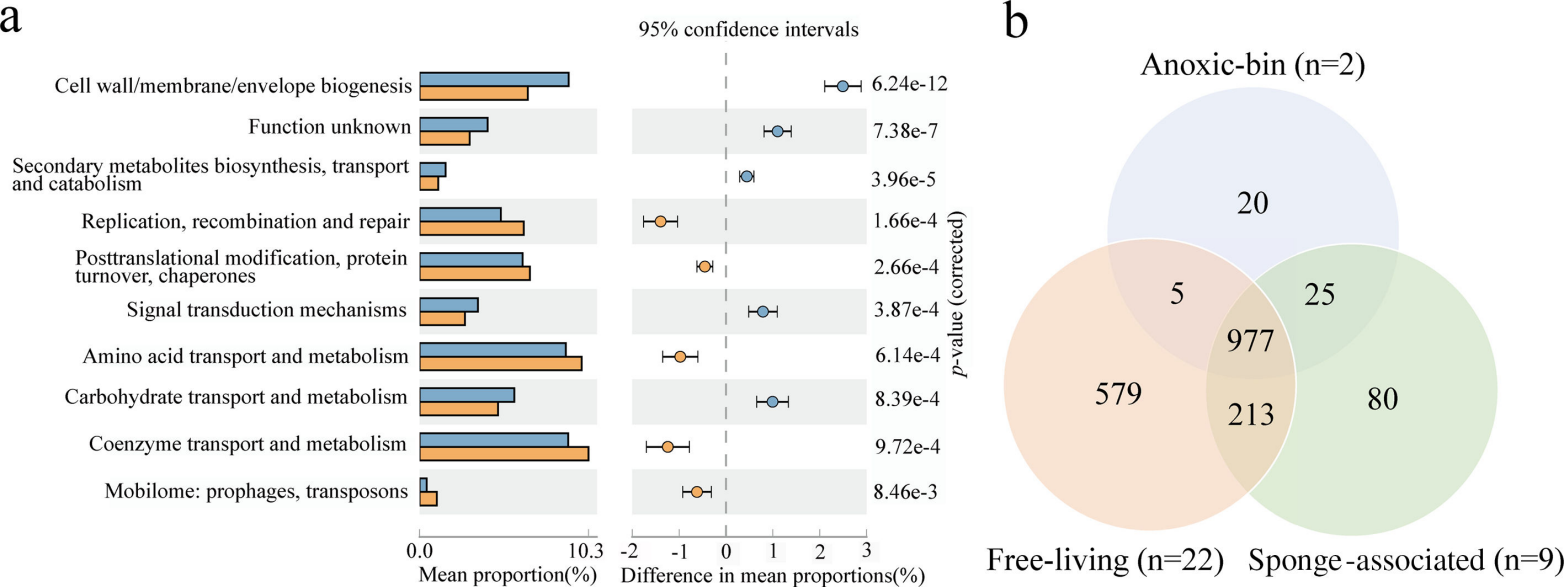
Difference analysis of the relative abundance of functional genes based on COG annotation. (**a**) COG classes with statistically significant differences in STAMP analysis between 9 sponge-associated genomes (orange) and 22 genomes of free-living cyanobacteria (blue). Error bars indicate within-group standard deviations. Presented categories passed a corrected *P* value of <0.05 in Welch’s *t*-test. (**b**) Venn diagram showing the number of COGs annotated for 22 free-living cyanobacterial genomes, 2 anoxia-adapted cyanobacterial genomes, and 9 sponge-symbiotic cyanobacterial genomes.

By comparing the genomes between the free-living and sponge-associated groups, we identified 10 COG categories that exhibited significant differences (Fig. 4a). The most significant difference was observed in the category of cell wall/membrane/envelope biogenesis (*P* < 0.05), with the free-living cyanobacteria having a higher number of annotated COGs in this category (Fig. 4a). Furthermore, the two *Synechococcus* from the anoxic layer shared 977 COGs with both the free-living and sponge-associated reference genomes, which represent their core functional proteins (Fig. 4b). Additionally, they possessed 20 unique COGs, of which 6 were associated with inorganic ion transport and metabolism, mostly related to ABC-type enterochelin transport systems involved in iron ion transport (COG4604, COG4605, COG4606, and COG4607).

Eukaryotic-like proteins are considered one of the markers for sponge-associated symbionts. The 34 genomes of cyanobacteria (Table S6) harbor ankyrin repeats (Ank), tetratricopeptide repeats (TPR), fibronectin type III domains (Fn3), cadherins (CAD), and WD40 repeats. Among them, TPR domains were ubiquitous, whereas Ank and Fn3 domains were restricted in free-living lineages (≤2 copies in rare instances). Conversely, Anoxic-bin48 and Anoxic-bin49 contained 15 and 14 Anks, respectively. Additionally, they exhibited a higher number of Fn3 domains, consistent with patterns observed in other “*Ca*. Synechococcus spongiarum” strains.

**Fig 5:**
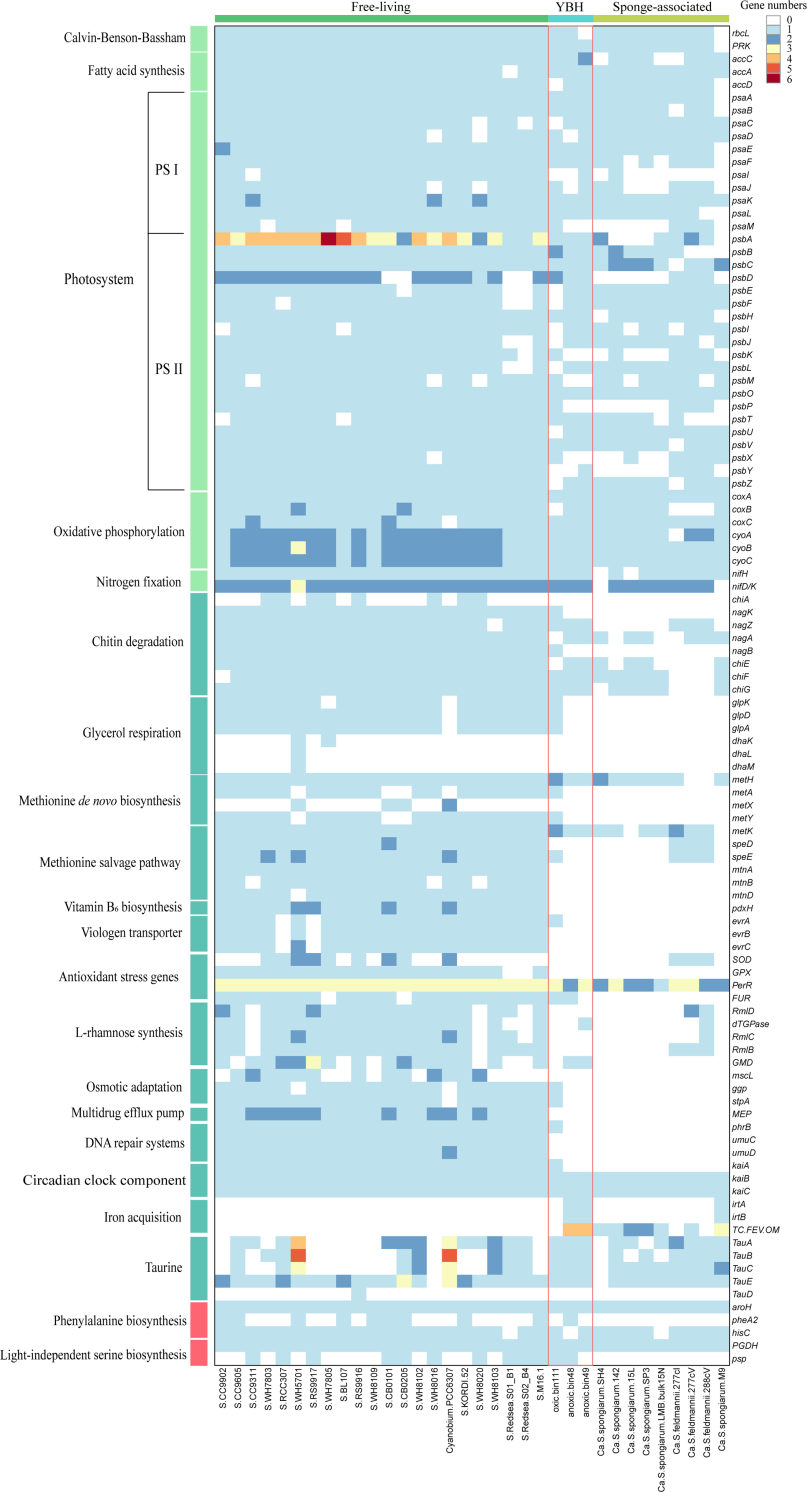
Heatmap illustrating the numbers of functional genes in 34 cyanobacteria MAGs. Heatmap showing the number of genes of the individual pathways in each genome.

The key genes (*rbcL* and *PRK*) involved in the complete Calvin-Benson-Bassham cycle for autotrophic metabolism and fatty acid synthesis were identified in all analyzed cyanobacterial genomes (Fig. 5). Genes related to photosynthesis were found in the free-living and sponge-associated *Synechococcus*. Specifically, genes encoding photosystem I, photosystem II, and the oxygen-evolving complex, including PsbP and PsbO, were present in three *Synechococcus* MAGs from YBH. The MAG for aerobic *Synechococcus* is associated with a lower module completeness, likely attributable to 70.43% of genome completeness. These results indicate that the genetic machinery for photosynthesis is well retained in all the MAGs. Transcriptomics analyses further showed that most photosynthesis-related genes of the MAGs were transcriptionally inactive under the dark, oxygen-depleted conditions, although limited transcription of individual components, such as *psbA,* was detected at 100 m depth (Table S7). In parallel, genes encoding cytochrome *c* oxidase (*coxABC*) and cytochrome *o* ubiquinol oxidase (*cyoABC*) were identified in three *Synechococcus* MAGs, indicating a retained genomic potential for aerobic respiration. However, the transcriptomic data revealed that these terminal oxidase genes were actively transcribed only in the Oxic-bin111, whereas no transcripts were detected in the Anoxic-bin48 and Anoxic-bin49 (Table S7). Nitrogen fixation is a key ecological function of many cyanobacteria that contribute significantly to ocean primary production and global nitrogen cycling. In YBH, the three reconstructed *Synechococcus* genomes all retain the canonical *nifHDK* gene cluster. However, transcriptional activity of nitrogen fixation was only detected in the oxic population Oxic-bin111, whereas no *nif* expression was observed in the Anoxic-bin48 and Anoxic-bin49 under current measurements (Table S7). This clear difference suggests that diazotrophy is active only in the oxygenated upper layer.

### Common features between anoxia-adapted and symbiotic *Synechococcus*

A subset of free-living *Synechococcus* possesses complete genetic pathways for chitin degradation (Fig. 5). In contrast, the anoxia-adapted and symbiotic lineages universally lack *chiA*, *nagK,* and *nagB* genes responsible for chitin utilization. Additionally, genes involved in glycerol respiration, including *glpK* (encoding glycerol kinase) and *glpD* (encoding membrane-associated glycerol-3-phosphate dehydrogenase), were identified in free-living *Synechococcus* genomes but were absent in the anoxia-adapted and symbiotic *Synechococcus* groups. Similarly, the free-living lineages uniquely retained the complete methionine salvage pathway, with genes *mtnA*, *mtnB*, and *mtnD* detected exclusively in these lineages but not identified in the anoxia-adapted and symbiotic *Synechococcus* genomes. Vitamin B_6_ biosynthesis capacity was also distinguished in the free-living lineages, as the *pdxH* gene (encoding pyridoxamine 5′-phosphate oxidase) was detected solely in these genomes but not in the anoxia-adapted or symbiotic *Synechococcus*.

Genome reduction in anoxia-adapted and symbiotic *Synechococcus* correlates with diminished environmental stress response capabilities. The *evrABC* genes, encoding permease components of the viologen exporter family transport system, were exclusively detected in the free-living cyanobacterial genomes. Their absence in the anoxia-adapted and symbiotic lineages indicates that they might inhabit slightly lower oxygen environments, thereby discarding certain defense mechanisms present in their free-living counterparts. Furthermore, SEED annotation of the cyanobacterial genomes through RAST analysis (23) revealed that the sponge-associated cyanobacteria have fewer antioxidant stress genes, compared to free-living counterparts (Table S8). Furthermore, the Anoxic-bin48 and Anoxic-bin49 were annotated with fewer antioxidant stress-related genes, compared to the other genomes, except for “*Ca*. S. spongiarum M9” and “*Ca*. S. spongiarum LMB bulk15N.” Specifically, the genomes of Anoxic-bin48 and Anoxic-bin49 lack genes encoding superoxide dismutase, glutathione peroxidase, peroxide stress regulator, and ferric uptake regulation protein, which are present in the free-living cyanobacteria. Extending to cell envelope biosynthesis, L-rhamnose, a component of O-antigens in lipopolysaccharides of gram-negative bacteria, has been previously detected in the marine *Synechococcus* of a free-living lifestyle (24). It is believed to play a role in host-microbe recognition and/or phage resistance. Proteins associated with rhamnose synthesis, including dTDP-4-dehydrorhamnose reductase (COG1091), dTDP-glucose pyrophosphorylase (COG1209), dTDP-4-dehydrorhamnose 3,5-epimerase (COG1898), dTDP-D-glucose 4,6-dehydratase (COG1088), and GDP-D-mannose dehydratase (COG1089), are typically absent in sponge-associated cyanobacteria (11). Only one gene (COG1089) for rhamnose synthesis was annotated in the genomes of Anoxic-bin48 and Anoxic-bin49 (Fig. 5) and was further confirmed by RASTtk analysis (25). Four proteins (COG1091, COG1209, COG1898, and COG1088) were annotated in the genomes of three facultative symbiotic “*Ca*. S. feldmannii” strains. All the other sponge-associated *Synechococcus* genomes lack the complete set of rhamnose synthesis genes. Consistent with this trend of reduced stress resilience in confined niches, genes critical for osmotic adaptation, including *mscL* (large conductance mechanosensitive channel), *ggp* (glucosylglycerol-phosphate synthase), and *stpA* (glucosylglycerol 3-phosphatase), were exclusively retained in the free-living cyanobacteria but absent in both anoxia-adapted and symbiotic lineages. Likewise, DNA repair systems countering UV damage (*phrB* and *umuCD*) and multidrug efflux pumps were uniquely present in the free-living cyanobacteria. Strikingly, the core circadian clock component *kaiA* gene was consistently identified in all the free-living lineages but was missing from the other *Synechococcus* genomes.

Conversely, there are also genes related to substance transport and utilization that are exclusively found in the *Synechococcus* genomes from anoxia-adapted and symbiotic environments. These lineages have likely developed convergent adaptations for iron acquisition: gene *irtA* (iron-regulated transporter) and TC.FEV.OM (which encodes an iron complex outer membrane receptor protein) were identified in the genomes of anoxia-adapted *Synechococcus* (Anoxic-bin48/49) and sponge symbionts, but not in the free-living lineages. The genomes of Anoxic-bin48 and Anoxic-bin49 also contain genes related to taurine transport, including genes encoding TauABCE proteins (COG0715, COG1116, COG0600, and COG0730), which may be involved in taurine transport. However, the *tauD* gene (COG2175), involved in taurine utilization by desulfonation to aminomaldehyde and sulfite, is present only in the genome of *Synechococcus* sp. RS9916, rather than the anoxia-adapted and symbiotic counterparts.

### Metabolic parallels between anoxia-adapted and free-living *Synechococcus*

Genomic analyses revealed that the anoxia-adapted *Synechococcus* might possess autonomous phenylalanine biosynthesis potential. The gene encoding chorismate mutase was universally identified across all the *Synechococcus* genomes. However, the *pheA2* gene encoding prephenate dehydratase, which converts prephenate to phenylpyruvate, was detected only in Anoxic-bin48 and the free-living *Synechococcus* but was absent in the symbiotic counterparts. The *hisC* gene encoding histidinol-phosphate aminotransferase, catalyzing the conversion of phenylpyruvate to phenylalanine, was identified in Anoxic-bin49 and the free-living *Synechococcus*. Both phosphoglycerate dehydrogenase and phosphoserine phosphatase (PSP) coding genes were detected in Anoxic-bin48. Notably, PSP was not encoded by the symbiotic *Synechococcus* genomes, while a subset of free-living *Synechococcus* genomes retained this gene.

## DISCUSSION

### The cyanobacteria in the anoxic layer of YBH are probably derived from a common ancestor with sponge-symbiotic *Synechococcus*

In this study, using metagenomic and metatranscriptomic data from YBH, three genomes of *Synechococcus* were obtained, representing two that survive in dark anoxic water layers. These two anoxia-adapted *Synechococcus* in YBH have a close phylogenetic relationship with “*Ca*. Synechococcus spongiarum M9” from the South China Sea geographically close to the location of YBH (21). However, “*Ca*. Synechococcus spongiarum M9” was revealed in a sponge collected from a well-illuminated, oxygenated marine habitat at a depth of 20 m (21). Although sponges were detected in YBH eukaryotic surveys (26), the microbial samples of this study were prefiltered to exclude large particles and eukaryotes, making direct host association unlikely. Hence, the anoxia-adapted *Synechococcus* in YBH was derived from a common ancestor with “*Ca*. Synechococcus spongiarum M9” and had undergone genomic variations to adapt to the permanently dark, anoxic environment of YBH. Currently, as far as we know, there are very few documented cases of active cyanobacteria in dark anoxic environments. Our transcriptomics evidence, combined with metagenomic abundance ranking and 16S rRNA gene amplicon data, confirms the existence of anoxia-adapted cyanobacteria in YBH.

“*Ca*. Synechococcus spongiarum,” a facultative symbiont, requires more ANK repeat sequences to avoid recognition by sponge cells. However, the opportunistic symbiont “*Ca*. Synechococcus feldmannii” utilizes a colonization strategy within sponge cells, requiring more FN3 and CAD domains with adhesive properties. The enrichment of FN3 domains suggests their involvement in adhesion and host colonization by attaching to glycoproteins, structural proteins, and CAD protein domains (27). Compared to other obligate symbionts, the cyanobacteria in the YBH anoxic layer have a higher number of FN3 domains, indicating that they might have a distinct symbiotic mode.

### Special metabolic potential and adaptation of *Synechococcus* in the anoxic layer

Regarding metabolic substrate utilization, our gene annotation showed that both anoxia-adapted and symbiotic cyanobacteria lack the chitin utilization genes *chiA* and *nagK*. Similarly, these genes are absent in low-light-adapted *Prochlorococcus* as a result of the evolutionary abandonment of chitin metabolic capacity (28). Cyanobacteria can employ extracellular glycerol as an additional carbon source for photomixotrophic chemical production; however, only free-living *Synechococcus* retains the key genes essential for the glycerol respiratory pathway. This restricted distribution is potentially linked to the pathway requirement for substantial oxygen or nitrate as a terminal electron acceptor (29). Taurine is a common sulfonic acid metabolite in marine sponges and serves as a critical intermediate in host-symbiont interactions (30). The presence of taurine transporters in the genomes of the *Synechococcus* in the anoxic layer suggests their capacity for taurine uptake. However, genes encoding canonical taurine cleavage pathways, including the oxygen-dependent taurine dioxygenase system and the oxygen-independent isethionate pathway (e.g., *tpa*, *tauF*, *iseH*, *iseG*) (31), were not identified in these genomes. In addition, the proposed respiration of sulfite derived from taurine to H₂S via DsrAB–DsrC is also unlikely to operate in the anoxia-adapted *Synechococcus* (32), as no homologs of *dsrAB* or *dsrC* were identified in these genomes. Whether there are alternative unknown taurine-degrading pathways in the anoxia-adapted *Synechococcus* remains to be answered.

*Synechococcus* in anoxic zones lacks the key gene involved in VB_6_ and methionine biosynthesis. This observation suggests that, akin to symbiotic *Synechococcus*, they likely acquire these essential compounds from external sources, such as the YBH heterotrophic microbial community (11).

Dark heterotrophic growth of cyanobacteria has indeed been documented in several laboratory model systems, including *Leptolyngbya boryana*, *Acaryochloris marina*, *Synechocystis* sp. PCC 6803, and *Anabaena variabilis* (33–36). In these studies, cyanobacteria were typically cultured under controlled conditions with supplemental organic carbon, and darkness or low-light exposure was transient or reversible. Cells generally retained a functional photosynthetic apparatus that remained inactive in the dark but could be rapidly reactivated upon re-exposure to light, often within hours. In contrast, the cyanobacterial MAGs recovered from the YBH originate from a naturally dark, permanently stratified, and persistently anoxic marine environment. Although genes encoding PSI, PSII, and the oxygen-evolving complex are retained in Anoxic-bin48 and -bin49, transcriptomics data indicate that most photosynthesis-related genes are transcriptionally silent under *in situ* conditions, with only *psbA* showing detectable transcription at the upper boundary of the anoxic zone. Consistently, in the permanently dark and anoxic Landsort Deep sediment samples, the *psbA* gene was highly abundant in the transcripts as well, even though its translation was halted in the darkness (37). This phenomenon may be the result of constitutive transcription and regulation of protein biosynthesis at the translation initiation level. Under anoxic and dark conditions, the transcription of the *psbA* gene may serve some unknown biological function unrelated to photosynthesis.

Likewise, genes encoding terminal oxidases involved in aerobic respiration are not transcriptionally active in the Anoxic-bin48 and -bin49. Together, these features distinguish the YBH cyanobacteria from previously described dark-grown or heterotrophically cultured cyanobacteria. Rather than representing a reversible physiological response to short-term darkness, the YBH anoxia-adapted cyanobacterial populations appear to occupy a long-term ecological niche characterized by sustained transcriptional inactivation of oxygen-dependent pathways. This natural system, therefore, extends existing models of cyanobacterial dark or heterotrophic growth by documenting genomic retention but persistent suppression of photosynthesis and aerobic respiration in a stable, light-deprived, and anoxic marine environment.

Regarding environmental stress responses, viologen transport proteins are typically involved in bacterial responses and resistance mechanisms against harmful compounds in the environment (38). Viologens can act as inhibitors of cyanobacterial photosynthesis, leading to increased oxidative stress and cell death (38). These proteins aid in the elimination of toxic viologen compounds, thereby reducing their intracellular concentrations and protecting cells from oxidative damage. Furthermore, reactive oxygen species (ROS) are byproducts of aerobic metabolism that can cause oxidative damage within cells. ROS generated by the photosynthetic electron transport chain, therefore, poses a threat to cyanobacteria (39). However, the anoxia-adapted *Synechococcus* in our study possesses fewer genes related to antioxidant stress. In dark and anoxic conditions of YBH, the production of peroxides significantly decreases due to the lack of oxygen, possibly leading to the loss of certain genes associated with antioxidant defense mechanisms in cyanobacteria. Similar observations have been reported in oxygen-depleted regions of the Bohai Sea, where both heterotrophic bacteria and the cyanobacteria showed a decrease in the number of antioxidant genes with decreasing dissolved oxygen concentrations (40). This suggests that in deoxygenated marine environments, microorganisms may reduce the number of antioxidant genes as an adaptation to oxygen deprivation. L-rhamnose is typically absent in sponge-associated cyanobacteria (11), potentially to avoid predation by the host. The identification of a gene encoding GDP-D-rhamnose synthase, which is crucial for rhamnose synthesis, implies that the anoxia-adapted *Synechococcus* of this study might exhibit facultative symbiotic characteristics (11). The relatively stable salinity in semi-enclosed water columns minimizes osmotic fluctuations for microorganisms. Consequently, the *Synechococcus* inhabiting the anoxic YBH zones has lost genes associated with osmotic stress tolerance. A significant deviation in the central axis occurs at 120 meter depth within this hole. Combined with progressive light attenuation through the water column, this results in complete darkness at the benthic zone. Accordingly, the *Synechococcus* in the dark, anoxic zone lacks the enzymatic systems required for repairing ultraviolet-induced DNA damage. Similarly, sponge symbiotic cyanobacteria do not maintain these repair functions due to the shelter effect of the host. The presence of genes encoding these proteins in the free-living cyanobacteria indicates a greater environmental pressure, potentially relying more on these proteins to withstand stressors. However, in sponge-symbiotic cyanobacteria, including those in the anoxic layer of YBH, the absence of these genes may be the result of co-evolution between the symbionts and their hosts. The Kai-based regulatory system orchestrates genome-wide transcription and diverse physiological processes in a circadian manner (41). The absence of light in benthic environments has thereby probably driven the loss of circadian regulation in the anoxia-adapted *Synechococcus* (42). Alternatively, the absence of KaiA may represent genomic streamlining, as *Synechococcus elongatus* retains dampened, low-amplitude oscillations with only KaiBC components preserved (43).

**Fig 6:**
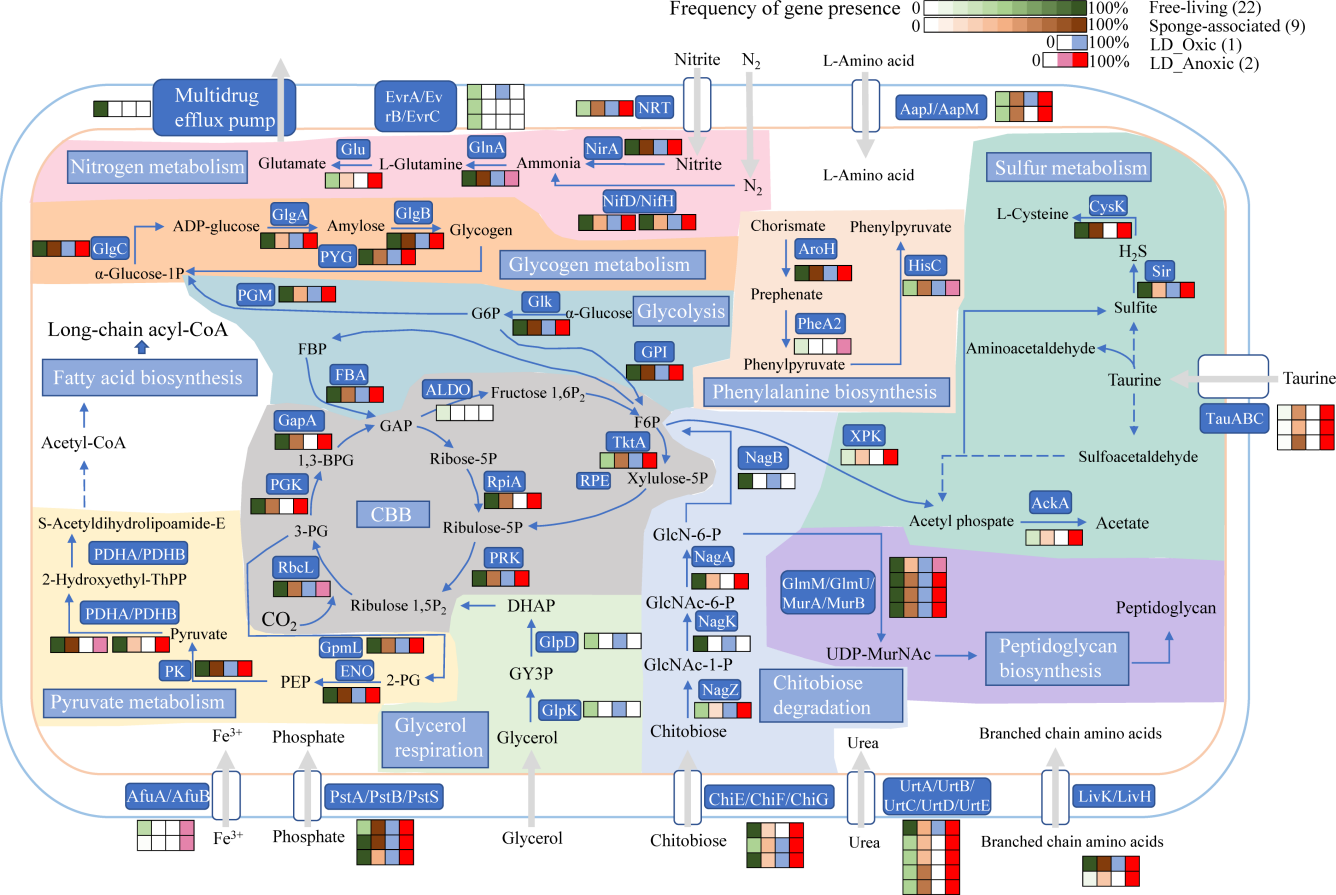
Metabolic reconstruction of major pathways in *Synechococcus* MAGs. The draft genomes for free-living and sponge-associated *Synechococcus* from NCBI were compared with the two MAGs from the anoxic YBH layer. The number or percentage of KEGG genes in these genomes was illustrated to mediate the metabolic network. α-Glucose-1P: α-Glucose-1-phosphate; G6P: α-D-Glucose 6-phosphate; F6P: D-Fructose 6-phosphate; FBP: D-Fructose 1,6-bisphosphate; 3-PG: 3-Phospho-D-glycerate; 1,3-BPG: 1,3-Bisphospho-D-glycerate; GAP: Glyceraldehyde 3-phosphate; Ribose-5P: Ribose 5-phosphate; Ribulose-5P: D-Ribulose 5-phosphate; Ribulose 1,5P_2_: D-Ribulose 1,5-bisphosphate; Fructose 1,6P_2_: D-Fructose 1,6-bisphosphate; Xylulose-5P: D-Xylulose 5-phosphate; PEP: Phosphoenolpyruvate; 2-PG: 2-Phospho-D-glycerate; DHAP: dihydroxyacetone phosphate. GY3P: glycerol-3-phosphate; GlcNAc-1-P: N-Acetyl-alpha-D-glucosamine 1-phosphate; GlcNAc-6-P: N-Acetyl-D-glucosamine 6-phosphate; GlcN-6-P: D-Glucosamine 6-phosphate.

In summary, the *Synechococcus* in the anoxic layer of YBH exhibits genomic features shared with sponge symbiotic cyanobacteria in oxic environments. However, they also possess characteristics associated with free-living cyanobacteria, which differ significantly from known facultative symbiotic cyanobacteria (Fig. 6). This suggests that the *Synechococcus* in the deep anoxic water of YBH has probably originated from some sponge symbiotic cyanobacteria and undergone long-term evolution to adapt to the conditions within the hole. With the expansion of oxygen-depleted zones, deoxygenation is expected to have negative impacts on the physiology, metabolism, and survival of many marine organisms (44). The presence of *Synechococcus* in the anoxic zone warrants further investigation, particularly the seasonal variations in their relative abundance. This could provide these organisms with a competitive advantage in future marine environments affected by climate change. Further research can be conducted to explore the adaptive mechanisms of prokaryotes and eukaryotes in the anoxic environment of the hole, to understand the evolutionary trends of species under oxygen-deprived stress in marine environments and to predict the diversity and evolutionary patterns in the hypoxic water column of similar geologically depressed areas and other open oceans.

### Conclusions

In this study, we identified actively transcribed cyanobacteria in the anoxic bottom layer of YBH. The anoxia-adapted cyanobacteria of YBH exhibited genomic features similar to those of sponge-associated cyanobacteria and contained a subset of functional genes for heterotrophs, suggesting the potential for a transition in their lifestyle. Therefore, this study uncovers a possible approach to the transformation of cyanobacteria between the environments of different light densities and oxygen levels. These findings also contribute to a better understanding of the emergence of new species in anoxic environments during the deoxygenation process in global marine waters, serving as a valuable research case for studying microbial evolution under oxygen-limited conditions.

## MATERIALS AND METHODS

### Study site, sampling procedures, and sequencing data

All the samples were collected from YBH in the South China Sea (16°31′30″N; 111°46'05″E). During two research cruises carried out in April and September of 2021, the water samples were collected at 21 different depths within YBH using the *in situ* microbial fixation and filtration (ISMIFF) apparatus (45). In the April cruise, 16 samples were collected in the depth range of 1–290 m, while in the September cruise, five samples were collected at depths of 96.5–100.5 m and 250 m (Table S1). The microorganisms were collected as previously described using the ISMIFF apparatus (14). The inlet of the ISMIFF was covered by a 30-μm pore size mesh. The DNA/cDNA libraries were prepared as mentioned in our previous study (14, 16), and then were sequenced using an Illumina Novaseq 6000 platform (2 × 150 bp). Temperature, salinity, and dissolved oxygen (DO) concentrations were measured *in situ* using environmental sensors (Fig. S1). Based on DO profiles, the water column in April was divided into an oxic layer (0–98 m, DO ≥ 2 mg/L), a suboxic layer (98–110 m, 0 < DO < 2 mg/L) (44), and an anoxic layer below 110 m. In September, the suboxic zone occurred at 75–101 m. Previous investigations conducted in different seasons and years have consistently documented a stable vertical dissolved oxygen stratification in the YBH (18, 19). Seasonal variability mainly causes minor vertical shifts in the oxycline rather than changes in the overall stratification pattern. Therefore, the lack of full seasonal replication is unlikely to substantially affect depth-resolved interpretations in this study.

### Quality control, assembly, and genome binning

The raw Illumina sequencing data from 21 metagenomes and 11 metatranscriptomes were trimmed and filtered by Fastp (v0.23.1) (46) with default settings, and then duplicate reads were removed using FastUniq (v1.1) (47). Clean data were decontaminated and co-assembled as mentioned in our previous study (16). 16S rRNA gene fragments for the V4 region (16S miTags) were extracted from the metagenomes (16). Eukaryotic contigs were filtered by using EukRep (v0.6.7) (48), and the remaining prokaryotic contigs with lengths longer than 2,000 bp were binned using metaWRAP (v1.3.2) (49). Completeness and contamination of the genomes were assessed using CheckM (v1.0.12) (50). The taxonomic placement of the MAGs was performed with GTDB-tk (v1.0.5) (51) using the GTDB (release207_v2) (52). Three MAGs belonging to cyanobacteria were retrieved from 21 metagenomes.

### Estimation of the relative abundance of genomes

The relative abundance of MAGs in metagenomes was calculated using the recruitment rates of the qualified reads mapped to the MAGs. The clean reads of metagenomes were aligned to all MAG contigs using BWA-MEM (v0.7.17) (53) with the default parameters and sorted by samtools (v1.9) (54). The “Relative Abundance” method in CoverM (v0.6.1, with setting -m relative_abundance --min-read-percent-identity 0.99 --min-read-aligned-percent 0.95 in genome mode) (https://github.com/wwood/CoverM) was used to calculate the relative abundances of MAGs in each of the metagenomes.

### Annotation and abundance profiling of functional genes

The coding sequences of each MAG were predicted and translated into protein sequences using Prodigal (v2.6.3) (55) with the setting “-p meta.” KEGG annotation was performed on all the proteins using KofamScan (v1.3.0) (56) and GhostKOALA (v2.0) (57), to assign KEGG Orthologs (KO) to the proteins using the KEGG release 105.0 database (58). Prediction of genomic ortholog clusters and PFAM domains was carried out using DIAMOND BLASTp (59) based on the COGs database (v2020) (22) and Pfam database (v36.0) (60), respectively. All the genomes were uploaded to the RAST (61) system for identification of open reading frames and their SEED annotations (23).

### Phylogenetic analysis of genomes

The 43 concatenated conserved protein sequences encoded by the genomes were extracted using CheckM (v1.0.12) (50). The protein sequences were aligned and trimmed using Mafft (v7.505) (62) and trimAl (v1.4) (63), respectively. The maximum likelihood phylogenetic tree was constructed using IQ-TREE 2 (v2.2.0) (64) with the following parameters: -m MFP -B 1000 -alrt 1000 -T AUTO. The resulting tree was visualized using iTOL v.6 (https://itol.embl.de/).

### Comparative genomics analysis

For the comparison of functional genes in the genomes, 31 representative bacterial genomes were downloaded from NCBI. These genomes comprised nine sponge-associated bacteria and 22 free-living bacterial genomes (Table S5). To assess the differences in the abundance of functional genes between the two bacterial groups, a two-tailed *t*-test was performed using STAMP v2.1.3 (65). Pairwise ANI values for the MAGs were calculated using ANIclustermap (https://github.com/moshi4/ANIclustermap). Moreover, the Bray-Curtis dissimilarity index calculated using the relative abundances of functional genes was used for PCoA.

## Data Availability

The assembly data of metagenome and metatranscriptome sequencing data and MAG sequences generated in this study have been deposited in the NCBI database under the accession code PRJNA900714.

## SUPPLEMENTAL MATERIAL

Supplemental figures aem.02576-25-s0001.docx Fig. S1 to S3.

Supplemental tables aem.02576-25-s0002.xlsx Tables S1 to S8.

ASM does not own the copyrights to Supplemental Material that may be linked to, or accessed through, an article. The authors have granted ASM a non-exclusive, world-wide license to publish the Supplemental Material files. Please contact the corresponding author directly for reuse.
