# Supplementary material for: Anoxia-adapted cyanobacteria in a marine blue hole
Source: Appl Environ Microbiol. 2026 Feb 23;92(3):e02576-25. doi: 10.1128/aem.02576-25 (PMC12997750; doi:10.1128/aem.02576-25)
Supplement: Supplemental figures — Fig. S1 to S3. [file aem.02576-25-s0001.docx]

# Supplementary material


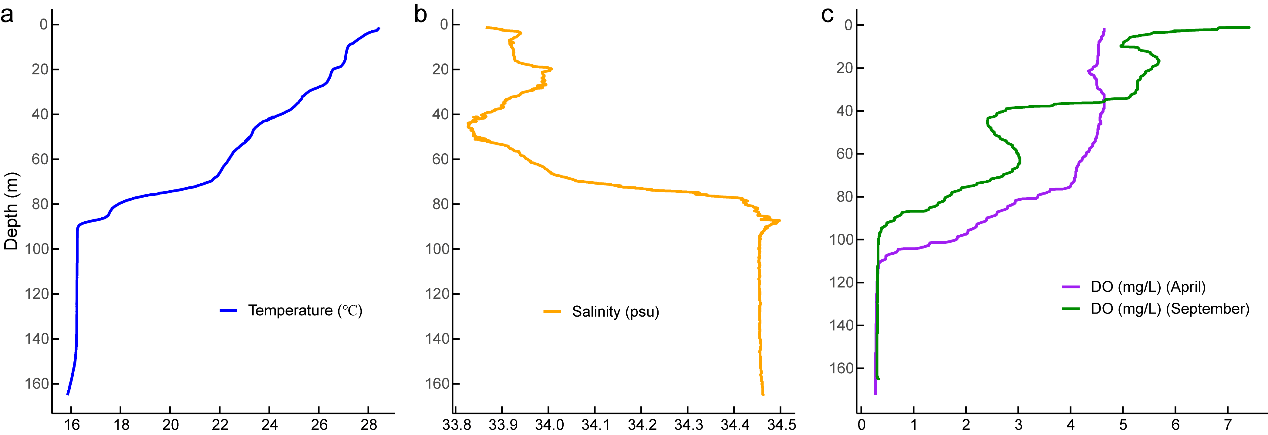


Supplementary Fig. 1 Vertical profiles of temperature, salinity, and dissolved oxygen in YBH waters.

a, Temperature (°C); b, Salinity; c, Dissolved oxygen (DO) (mg/L). DO concentrations were measured in April and September.


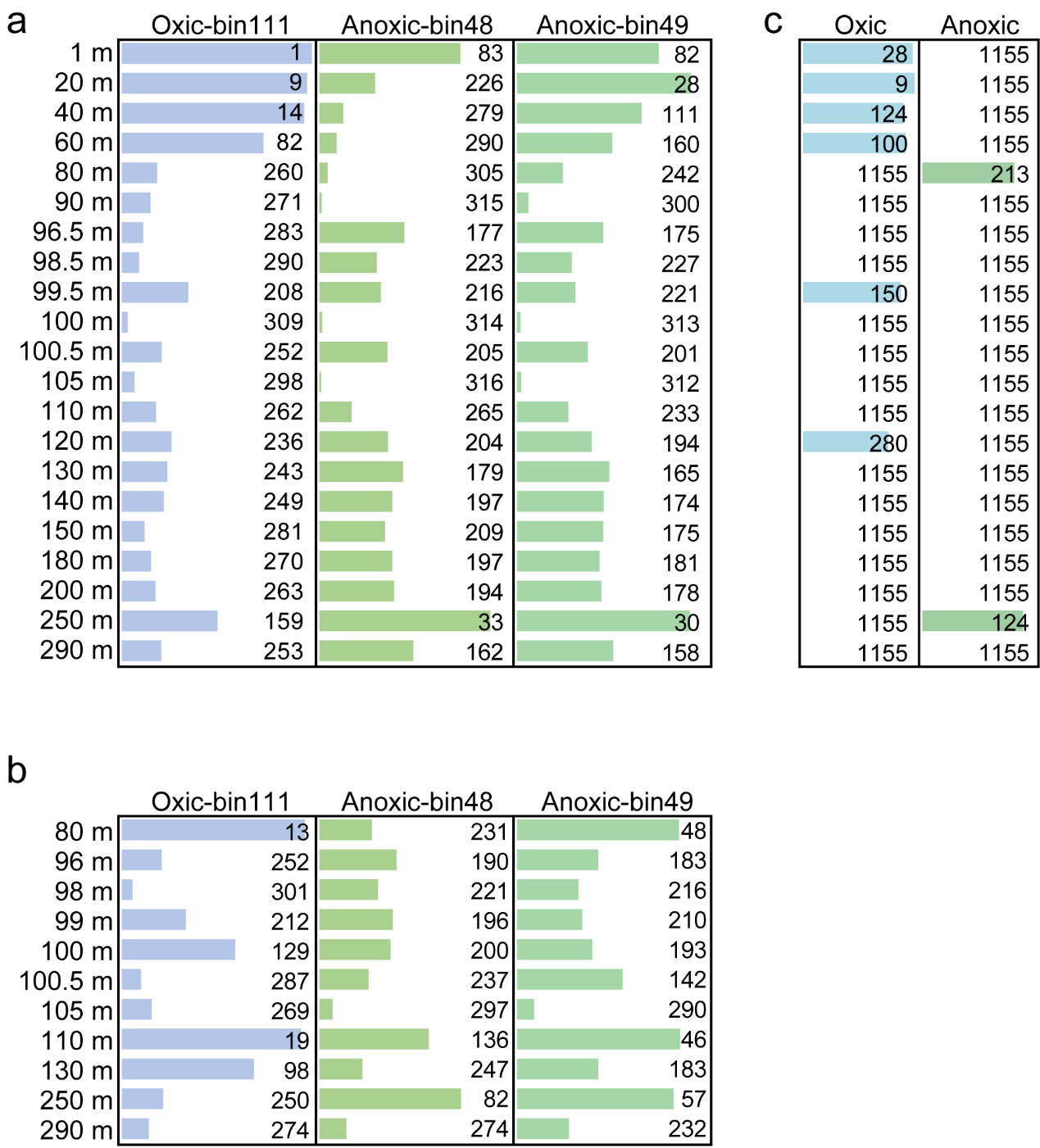


Supplementary Fig.2 Relative abundance ranking of the three cyanobacterial MAGs in metagenomic and metatranscriptomic data.

a, Relative abundance ranking of the three cyanobacterial MAGs across the 21 metagenomes; b, Relative abundance ranking of the three cyanobacterial MAGs across the 11 metatranscriptomes; c, Relative abundance ranking at the genus level of the 16S sequences affiliated with the three cyanobacterial MAGs within the microbial community.


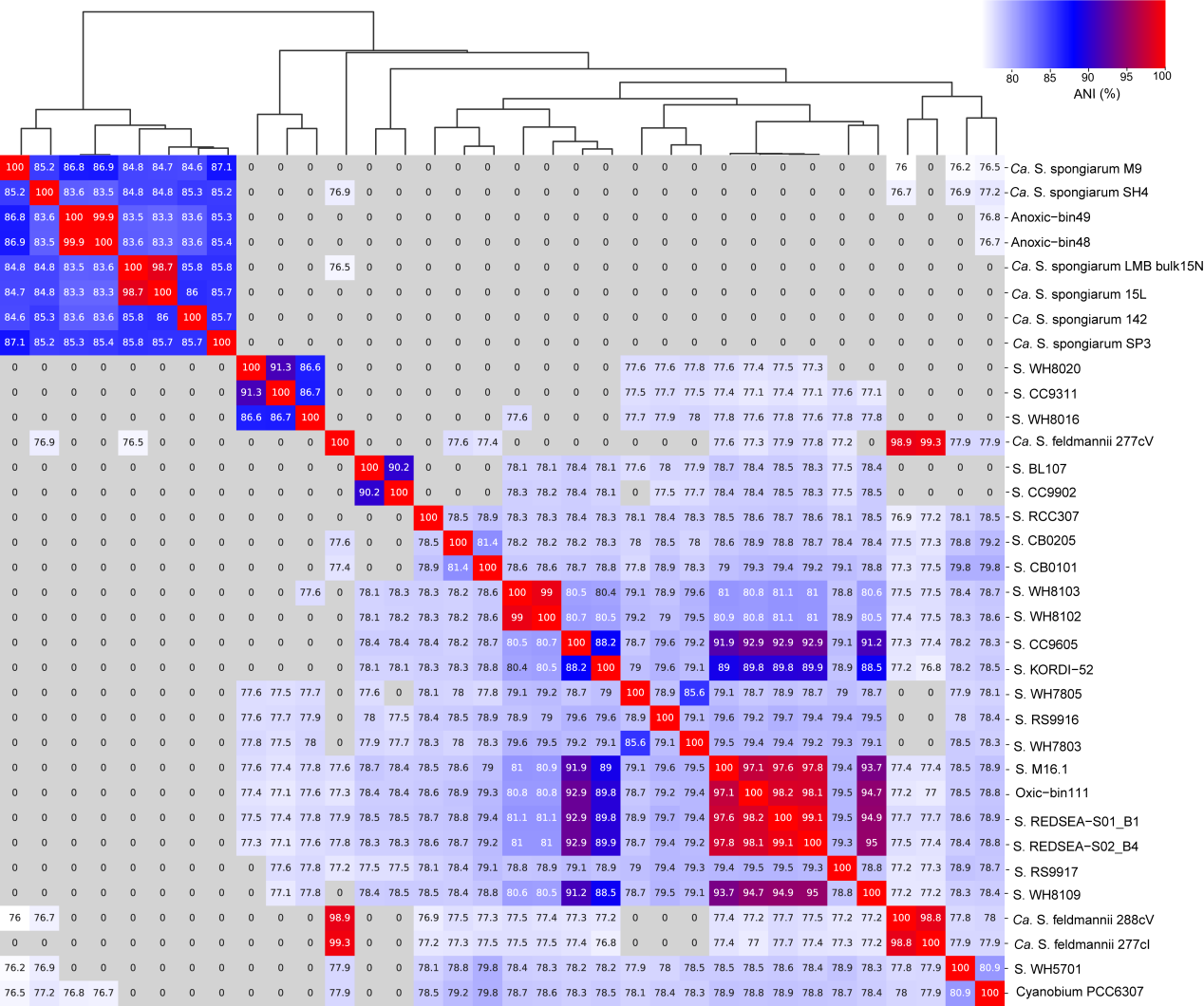


Supplementary Fig. 3 Pairwise ANI of cyanobacterial MAGs.

Anoxic-bin48 and Anoxic-bin49 are binned from the metagenomes for the anoxic layer of the Yongle blue hole. All genomes of *Candidatus* Synechococcus spongiarum (*Ca.* S. spongiarum) were downloaded from the NCBI database.
